# Inhibition of Endothelial Cell Tube Formation by Anti-Vascular Endothelial Growth Factor/Anti-Angiopoietin-2 RNA Nanoparticles

**DOI:** 10.3390/pharmaceutics17010055

**Published:** 2025-01-03

**Authors:** Cheng Zhong, Zhanquan Shi, Chia-Yang Liu, Daniel W. Binzel, Kai Jin, Xin Li, Peixuan Guo, S. Kevin Li

**Affiliations:** 1 Division of Pharmaceutical Sciences, James L Winkle College of Pharmacy, University of Cincinnati, Cincinnati, OH 45267, USA; 2 Department of Ophthalmology, College of Medicine, University of Cincinnati, Cincinnati, OH 45267, USA; 3 Center for RNA Nanobiotechnology and Nanomedicine, College of Pharmacy, James Comprehensive Cancer Center, The Ohio State University, Columbus, OH 43210, USA; 4 College of Medicine, The Ohio State University, Columbus, OH 43210, USA

**Keywords:** nanotechnology, RNA nanoparticle, anti-angiogenesis, 3D spheroid sprouting assay

## Abstract

RNA nanoparticles, derived from the packaging RNA three-way junction motif (pRNA-3WJ) of the bacteriophage phi29 DNA packaging motor, have been demonstrated to be thermodynamically and chemically stable, with promise as a nanodelivery system. **Background/Objectives**: A previous study showed that RNA nanoparticles with antiangiogenic aptamers (anti-vascular endothelial growth factor (VEGF) and anti-angiopoietin-2 (Ang2) aptamers) inhibited cell proliferation via WST-1 assay. To further investigate the antiangiogenic potential of these RNA nanoparticles, a modified three-dimensional (3D) spheroid sprouting assay model of human umbilical vein endothelial cells was utilized in the present study. **Methods**: Three groups of RNA nanoparticles were evaluated, namely, pRNA-3WJ series, RNA square series (polygon-type RNA nanoparticles), and 8WJ series (multiple-way junction RNA nanoparticles), which were conjugated with a single anti-VEGF, the combination of one anti-VEGF and one anti-Ang2, or multiple anti-VEGF aptamers. The core scaffold RNA nanoparticles (without aptamers) were used as the references, and bevacizumab was used as the positive control. **Results**: The results demonstrated the inhibition effects of the RNA nanoparticles on endothelial cell tube formation at 67 nM in a 3D spheroid sprouting model. The results in the 3D spheroid sprouting assay are consistent with those of the WST-1 proliferation assays. **Conclusions**: Among the RNA nanoparticles evaluated, 3WJ-3VEGF and SQR-VEGF-Ang2 had inhibition effects equivalent to bevacizumab and were promising for anti-angiogenesis treatment.

## 1. Introduction

Angiogenesis is a fundamental process during development but can also contribute to the pathology of various diseases such as cancers and ophthalmic diseases. Many regulators are involved in angiogenesis, such as vascular endothelial growth factor (VEGF) and angiopoietin-2 (Ang2) [1]. VEGF is one of the vital and well-studied growth factors in angiogenesis, and Ang2 has been suggested to enhance the angiogenic effects of VEGF in angiogenesis [1,2,3]. As VEGF plays a central role in angiogenesis, numerous therapeutic approaches targeting VEGF, such as aptamers, oligonucleotides, and monoclonal antibodies, have been studied and developed [4]. Among them, bevacizumab (Avastin) and pegaptanib (Macugen) were the first two anti-VEGF drugs approved in 2004 [5]. Bevacizumab, a recombinant human VEGF monoclonal antibody, has been approved in a range of solid tumor indications and as an important part of the standard of care in oncology [5,6]. It has also been used as an off-label treatment for wet age-related macular degeneration (AMD) and diabetic retinopathy [7]. Approved in the same year, pegaptanib was the first VEGF inhibitor for the treatment of wet AMD, and it was the first available RNA-based aptamer approved for therapeutic use [8,9].

RNA-based therapeutic agents are promising in disease treatment [10,11]. However, RNA is relatively unstable at ultra-low concentration and prone to degradation by ribonucleases after administration into the body [11]. Therefore, most RNA-based therapies require a high concentration of these agents to achieve therapeutic effects. To overcome it, RNA-based therapeutic agents are chemically modified during synthesis to increase their resistance to nucleases without reducing activity [11]. For example, the chemical modifications in pegaptanib include 2′-O-methyl, 2′-fluoro, and PEG conjugation [9,10,11]. Another strategy is utilizing a delivery carrier to efficiently transport RNA-based agents to their targets through extracellular and intracellular barriers [11]. In our previous study, anti-VEGF aptamers were conjugated into RNA nanoparticles served as a carrier for posterior eye delivery, and the antiangiogenic effects of RNA nanoparticles containing anti-VEGF aptamers were demonstrated on endothelial cells using a WST-1 cell proliferation assay [12].

The packaging RNA three-way junction motif (pRNA-3WJ) of the bacteriophage phi29 DNA packaging motor, with the ability to fold into a compact structure that constituted an RNA nanoparticle, was previously discovered [13,14]. The pRNA-3WJ (3WJ), as a basic core scaffold, can be used to construct multiple types of RNA nanoparticles: (a) multiple-way junction RNA nanoparticles redesigned from 3WJ, such as RNA 4-way junction (4WJ), RNA 6-way junction (6WJ), and RNA 8-way junction (8WJ); and (b) polygon-type RNA nanoparticles of different sizes and shapes using 3WJ as the polygon corners by turning its angle, such as RNA triangle, RNA square with tunable sizes (length of 5, 10, or 20 nm each side), and RNA pentagon [15]. These RNA nanoparticles were shown to be thermodynamically and chemically stable after chemical modification such as 2′-fluoro modification [13]. Since RNA nanoparticles have defined size, structure, and stoichiometry, they have known and controllable characteristics, with the benefits of reproducibility during manufacturing. Multiple functional agents, such as RNA aptamer, miRNA, and therapeutic siRNA, can be added to the core scaffold of the nanoparticle as subunits [16]. The RNA nanoparticles with RNA aptamers can provide superior specificity to receptors and have lower antibody-inducing activity than protein-based therapeutics [17]. For example, the delivery of miRNA and paclitaxel by 6WJ RNA nanoparticles can inhibit cancer growth in liver cancer xenografts [18]. pRNA-3WJ can deliver EGFR targeting RNA aptamers and siRNA to inhibit tumor growth in KRASG12C non-small cell lung cancer tumor xenografts [19]. These developments have demonstrated that anti-VEGF aptamers can be conjugated to RNA nanoparticles to improve their stability and biodistribution for potential anti-angiogenesis and anti-tumor treatments.

Our previous study demonstrated the antiangiogenic potential of RNA nanoparticles with anti-VEGF aptamers using the WST-1 cell proliferation assay [12], a two-dimensional (2D) assay. The 2D cell proliferation assay, based on cell counting, is rapid, reproducible, and reliable in evaluating the effects of test agents on angiogenesis. However, it is limited to proliferation rates and can be interfered with by both cell division and death, which are difficult to distinguish and exclude simultaneously [20]. Therefore, an alternative method of investigating the antiangiogenic potential of RNA nanoparticles and supporting the results obtained in the WST-1 cell proliferation assay is beneficial.

The three-dimensional (3D) spheroid sprouting assay, originally established and developed by Korff and Augustin [21,22], has been demonstrated to be suitable to screen proangiogenic and antiangiogenic agents [23,24]. Compared to the 2D proliferation assay, the 3D spheroid sprouting assay involves the cell migration, proliferation, and sprouting processes, providing a three-dimensional cell–cell matrix and a cell–extracellular matrix, which closely mimic the in vivo situation of angiogenesis [20,25]. Therefore, this assay was used to identify and evaluate the antiangiogenic potential of RNA nanoparticles in the present study. The objectives of this study were (a) to use the 3D spheroid sprouting assay to evaluate the potential antiangiogenic effects of RNA nanoparticles in a complex environment; (b) to compare the results from the 3D assay to those of 2D WST-1 proliferation assay; and (c) to compare the inhibitory effects of RNA nanoparticles on endothelial cell tube formation in the assay to identify the nanoparticles with the most effects among the studied nanoparticles. Three groups of RNA nanoparticles containing anti-VEGF and anti-Ang2 aptamers, including 3WJ series, RNA square (SQR) series, and 8WJ series, were evaluated for their antiangiogenic potential and compared in this study: (a) 3WJ, as the basic RNA nanoparticles, was conjugated with single or multiple anti-VEGF aptamers; (b) SQR, one of the polygon-type RNA nanoparticles, was conjugated with a single anti-VEGF aptamer or the combination of one anti-VEGF aptamer and one anti-Ang2 aptamer; and (c) 8WJ, one of the multiple-way junction RNA nanoparticles, was conjugated with a single anti-VEGF aptamer.

## 2. Materials and Methods

### 2.1. Materials

EGM^TM^-2 MV microvascular endothelial cell growth medium-2 BulletKit^TM^, containing EBM^TM^-2 basal medium and EGM^TM^-2 MV microvascular endothelial cell growth medium SingleQuots^TM^ supplements, was purchased from Lonza (Walkersville, MD, USA). Human VEGF-165 recombinant protein was purchased from Thermo Fisher Scientific (Waltham, MA, USA). Methyl cellulose (4000 centipoises) was purchased from Sigma-Aldrich (St. Louis, MO, USA). Fisherbrand^TM^ petri dish (100 mm) was purchased from Fisher Scientific (Florence, KY, USA). Gibco^TM^ Collagen I, rat tail (3 mg/mL), was purchased from Fisher Scientific (Pittsburgh, PA, USA) and diluted with 0.1% acetic acid to 2 mg/mL. Medium 199 (10X) was purchased from Sigma-Aldrich (St. Louis, MO, USA). Bevacizumab (Avastin, 25 mg/mL) was purchased from Besse Medical (West Chester, OH, USA). Paraformaldehyde (PFA) was purchased from Thermo Fisher Scientific (Waltham, MA, USA) for the preparation of 10% (*w*/*v*) PFA in PBS and adjusted to pH 7.4.

### 2.2. RNA Nanoparticles

**Figure 1:**
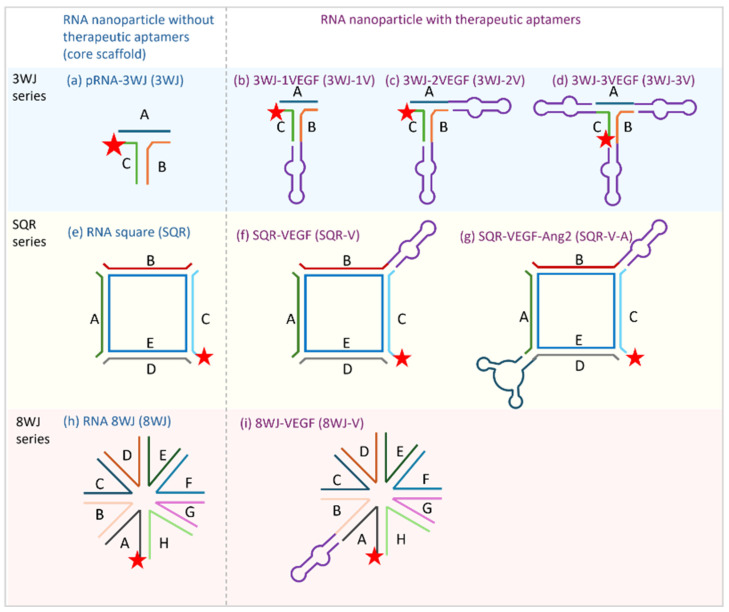
Tested RNA nanoparticles and their structures: (**a**) pRNA-3WJ; (**b**) 3WJ-1V; (**c**) 3WJ-2V; (**d**) 3WJ-3V; (**e**) RNA square; (**f**) SQR-V; (**g**) SQR-V-A; (**h**) RNA 8WJ; and (**i**) 8WJ-V. The lines with letters (A–H) represent the different RNA sequences; the same sequences are marked with the same color in each series. The stars represent the Alexa 647 label. The purple loop subunit represents the anti-VEGF aptamer. The dark blue loop subunit represents the anti-Ang2 aptamer.

RNA nanoparticles were either purchased from ExonanoRNA (Columbus, OH, USA) or synthesized via a self-assembly process based on the design of the RNA sequences [13]. The stability and properties of the RNA nanoparticles were assessed as described previously [26]. Figure 1 displays the RNA nanoparticles evaluated in the present study and their structural information. Table 1 lists the RNA sequences of the RNA nanoparticles. Three groups of RNA nanoparticles were investigated: (a) pRNA-3WJ and its derivatives; (b) RNA square and its derivatives; and (c) RNA 8WJ and its derivative. The basic RNA nanoparticles in each system were the core scaffold that had different shapes and sizes. The small size nanoparticle pRNA-3WJ (3WJ, Figure 1a) was the basic RNA nanoparticle. It was used as a corner to construct a larger square-shaped nanoparticle with 10 nm side length, i.e., RNA square (SQR, Figure 1e). The RNA 8WJ (8WJ, Figure 1h) was a larger nanoparticle redesigned from 3WJ. The core scaffold nanoparticles were the carriers. These core scaffold nanoparticles were conjugated with single or multiple anti-VEGF aptamers and an anti-Ang2 aptamer as the therapeutic modules to form the angiogenesis-inhibiting RNA nanoparticles. The RNA nanoparticles were labeled with Alexa Fluor 647 (the fluorescent label was used for visualization in the previous pharmacokinetics study [12] but was not involved in the present study). The experimental RNA nanoparticle solutions were freshly prepared by diluting the stock solutions from −20 °C storage with EBM^TM^-2 basal medium to the working concentration, 67 nM, for the HUVEC spheroid sprouting assay.

### 2.3. Cell Culture

Primary human umbilical vein endothelial cells (HUVEC) were purchased from ATCC (Manassas, VA, USA). HUVEC was cultured in EBM^TM^-2 basal medium supplemented with several growth factors in EGM^TM^-2 MV kit at 37 °C in 5% CO_2_ until passage 4. HUVEC of passage 5 was used for the 3D spheroid sprouting assays.

### 2.4. Three-Dimensional Spheroid Sprouting Assay

#### 2.4.1. Spheroid Generation

Upon reaching confluence of HUVEC at passage 4, the cells were calculated to form 500 cells/spheroid and suspended in a medium solution prepared by mixing, in volume, 4 parts limited-nutrient medium and 1 part methocel stock solution. The limited-nutrient medium was EBM^TM^-2 basal medium supplemented with 0.5% fetal bovine serum (FBS, Lonza) and 30 ng/mL VEGF, which is consistent with the limited-nutrient medium used in our previous 2D WST-1 proliferation assay [12]. Methocel stock solution was prepared as EBM^TM^-2 basal medium containing 1.2% methyl cellulose [27]. Spheroids were generated by hanging cell drops [27]. Briefly, 30 µL of cell suspension in the mixture above was dropped in the petri dish. The drops were then incubated upside-down by inverting the petri dish at 37 °C in 5% CO_2_ for 24 h to form spheroids.

#### 2.4.2. Spheroid Sprouting Assay

After the spheroids formed, they were collected and resuspended in the collagen matrix solution by mixing 1 part methocel solution containing 4% FBS and 1 part collagen medium at 4 °C. The collagen medium was prepared at 4 °C in advance by mixing 8 parts 2 mg/mL collagen, 1 part 10X medium M199, and approximately 1.2 parts 0.2 N NaOH to adjust the pH until the color of pH indicator in the collagen medium changed from yellow to orange. The spheroids embedded in the collagen matrix solution were then immediately transferred to the 24-well plates (0.5 mL containing ~40–50 spheroids in each well) and were incubated at 37 °C in 5% CO_2_ for 30 min for gel polymerization. After the gels were formed, 100 µL EBM^TM^-2 basal medium containing 0.4 µM RNA nanoparticles (final concentration as 67 nM; all concentrations mentioned below are final concentrations) or the other tested reagents were added on top of the gels in each well. The spheroids treated with 67 and 333 nM (10 and 50 µg/mL) bevacizumab were the positive controls, while the spheroids treated with EBM^TM^-2 basal medium only were the negative control (basal spheroids), providing the baseline sprouting activity of the spheroids. To confirm that the assay was sensitive and versatile to both antiangiogenic and proangiogenic reagents and feasible for the RNA nanoparticle evaluation, two groups of treatments were tested first: (a) bevacizumab at 67 and 333 nM as the inhibitor and (b) VEGF at 0.008, 0.031, 0.125, and 0.5 nM as the stimulus. The spheroids were incubated for 24 h in the treatments. After that, 0.6 mL 10% PFA was added to each well to stop the sprouting assay.

To evaluate the effect of the treatments, images of the spheroids were acquired via an inverted microscope with a 10× objective lens (Leica TCS SPE, Leica Microsystems, Wetzlar, Germany) and processed and analyzed by AxioVision SE64 Rel. 4.8 software (Carl Zeiss Microscopy, White Plains, NY, USA). At least 10 spheroids in each well were randomly selected and imaged. The length of all sprouts of each spheroid were measured manually, and the cumulative sprout length (CSL) was calculated to quantitively evaluate the angiogenesis of the spheroids. In addition to the CSL analysis, the numbers of sprouts per spheroid and the numbers of branching points per spheroid in the images were counted in selected treatments for comparison. The sizes of these spheroids were also measured in the images. Three biological replicates were conducted, and the data were combined and analyzed.

### 2.5. Comparison Between 3D and 2D Assays

As part of the continuing pharmacodynamics evaluation of the nanoparticles, the nanoparticle data obtained from the 2D WST-1 proliferation assay of HUVEC were compared to the results from the 3D HUVEC spheroid spouting assay in the present study. The nanoparticles were tested at 100 nM in the 2D model, and the data were normalized to calculate the percentage effect based on the positive control at the same concentration of nanoparticles [12]. The percentage effect (%*Effect_2D_*) was calculated using the absorbance of cells in the WST-1 assay after treatment with RNA nanoparticles (100 nM) and those of the negative (the medium without the nanoparticles) and positive controls (bevacizumab, 100 nM) in each set:

(1) $${\%Effect}_{2D}=\frac{A_{negative}-A_{nano}}{A_{negative}-A_{positive}}\times 100\%$$

where *A_negative_* is the absorbance of cells in the negative control group, *A_nano_* is the absorbance of cells treated with RNA nanoparticles, and *A_positive_* is the absorbance of cells treated with bevacizumab at the same concentration of the nanoparticles within the model. To be consistent with the 2D assay, the CLS data were also normalized to calculate the percentage effect (%*Effect_3D_*) based on the positive control of the same concentration of bevacizumab using the CLS of spheroids treated with RNA nanoparticles and those of the negative (basal) and positive controls in each replicate:

(2) $${\%Effect}_{3D}=\frac{{CLS}_{base}-{CLS}_{nano}}{{CLS}_{base}-{CLS}_{positive}}\times 100\%$$

where *CLS_base_* is the CLS of spheroids in the negative/basal control group, *CSL_nano_* is the CLS of spheroids treated with RNA nanoparticles, and *CLS_positive_* is the CLS of spheroids treated with bevacizumab at the same concentration of the nanoparticles within the model. The percentage effects (%Effect) were used to compare the inhibition effect of the RNA nanoparticles on cell proliferation using bevacizumab at the same concentration as the reference. For example, the %Effect of 100% would indicate that the RNA nanoparticles had the same spheroid tube formation inhibitory effect as bevacizumab. The %Effect results from the 3D and 2D assays were compared.

### 2.6. Statistical Analysis

The data obtained in the present study were processed and presented using Microsoft Excel version 2410 (Redmond, WA, USA). The highest and lowest data points in each treatment group were discarded to calculate the truncated mean and then presented as the truncated mean ± standard error of the mean (SEM). Statistical analyses including *t*-tests and one-way ANOVA were performed with GraphPad Prism version 10.3.1 (Boston, MA, USA).

## 3. Results

### 3.1. Feasibility of 3D Spheroid Sprouting Assay to Evaluate the Nanoparticles

**Figure 2:**
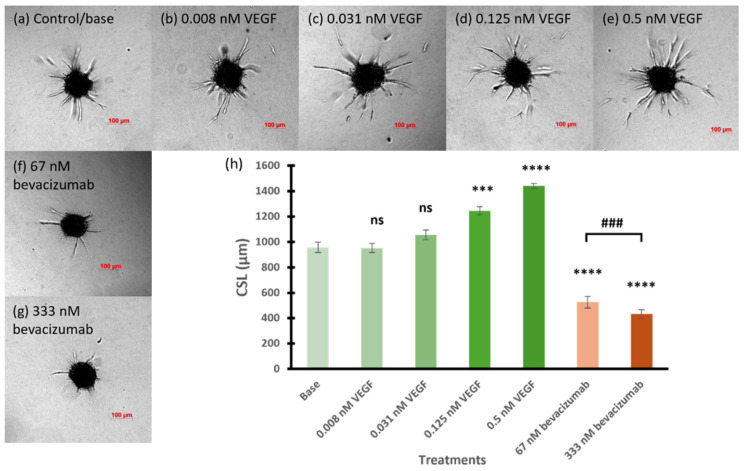
Representative images of (**a**) base spheroids, (**b**–**e**) spheroids treated with multiple concentrations of VEGF, and (**f**,**g**) spheroids tested with bevacizumab at 67 and 333 nM, respectively. (**h**) The cumulative sprout length of spheroids treated with multiple concentrations of VEGF and bevacizumab. Truncated mean ± SEM (n = 29–36). The symbol * indicates *t*-tests between the specific treatment and base spheroids (control without treatment), ns *p* > 0.05, *** *p* < 0.001, **** *p* < 0.0001; # indicates *t*-test between two specific treatments, ### *p* < 0.001.

Before evaluating the effects of RNA nanoparticles, bevacizumab at two concentrations 67 and 333 nM were used as positive controls to inhibit angiogenesis and to assess the feasibility of the 3D spheroid sprouting assay in the testing. In comparison, VEGF, at concentrations of 0.008, 0.031, 0.125, and 0.5 nM, was used to promote angiogenesis as another reference to examine the sensitivity and versatility of the 3D in vitro model. Figure 2 shows the representative images and CSL of the spheroids promoted with multiple concentrations of VEGF or inhibited with bevacizumab. The proangiogenic effect of the VEGF on the CSL of the spheroids increased with the increase in its concentration. Increases in the number and length of the sprouts and in the number of branching points were observed in the spheroids promoted with increasing concentrations of VEGF (Figure 2b–e). Starting from 0.031 nM, VEGF slightly elicited the angiogenic sprouting of the spheroids with a CSL of 1056 µm, compared to the baseline CSL of 957 µm. The CLS values of spheroids treated with 0.125 and 0.5 nM VEGF were significantly increased to 1246 and 1441 µm, respectively, compared to those of basal spheroids (*p* < 0.001 for 0.125 nM and *p* < 0.0001 for 0.5 nM). Conversely, the spheroids treated with the angiogenesis-inhibiting drug, bevacizumab, at 67 and 333 nM, exhibited a decrease in sprouting ability in terms of both sprout number and length (Figure 2f,g). Bevacizumab, at 67 nM, had a significant inhibitory effect on the sprout formation of the spheroids with the CSL of 525 µm (*p* < 0.0001 compared to basal spheroids). When the concentration of bevacizumab increased to 333 nM, the spheroids showed a significantly decrease in sprouting activity compared to those treated with basal medium and 67 nM bevacizumab (*p* < 0.0001 and *p* < 0.001, respectively). These results confirmed the sensitivity of the present 3D model and demonstrated its utility to evaluate the effects of the antiangiogenic RNA nanoparticles.

### 3.2. Inhibitory Effect of RNA Nanoparticles

The antiangiogenic potential of the RNA nanoparticles was evaluated via the 3D spheroid sprouting assay. Three groups of RNA nanoparticles at 67 nM, the same concentration as that of bevacizumab (positive control), were examined: 3WJ with single or multiple anti-VEGF aptamers (3WJ-1V, 3WJ-2V, and 3WJ-3V, Figure 1b–d); SQR with a single anti-VEGF aptamer or a combination of one anti-VEGF aptamer and one anti-Ang2 aptamer (SQR-V and SQR-V-A, Figure 1f,g); and 8WJ with a single anti-VEGF aptamer (8WJ-V, Figure 1i). The core scaffolds—3WJ, SQR, and 8WJ—without the aptamers were used as references and were compared to the basal spheroids (the negative control).

#### 3.2.1. 3WJ Series

**Figure 3:**
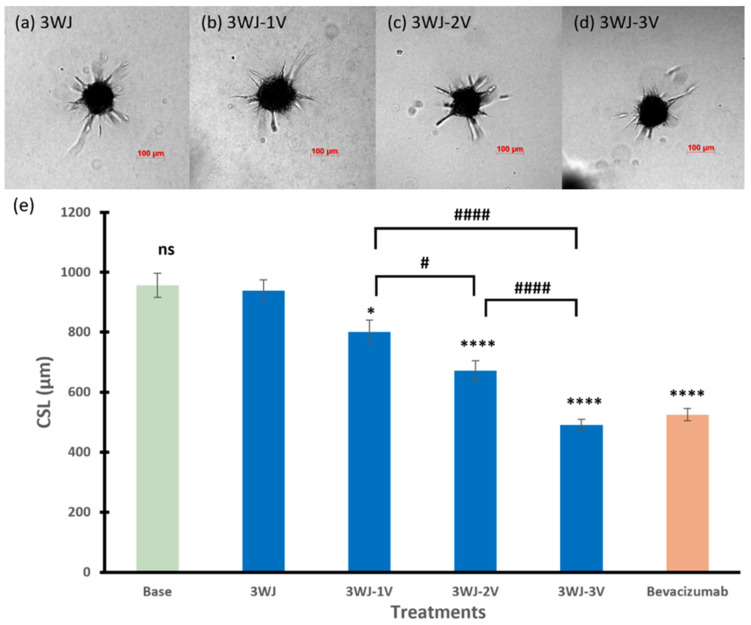
Representative images of spheroids treated with (**a**) 3WJ, (**b**) 3WJ-1V, (**c**) 3WJ-2V, and (**d**) 3WJ-3V. (**e**) The cumulative sprout length of spheroids treated with RNA nanoparticles in 3WJ series. Truncated mean ± SEM (n = 29–36). The symbol * indicates *t*-tests between the specific treatment and core scaffold RNA nanoparticle 3WJ, ns *p* > 0.05, * *p* < 0.05, **** *p* < 0.0001; # indicates *t*-tests between two specific treatments, # *p* < 0.05, #### *p* < 0.0001.

Figure 3 summarizes the effects of 3WJ series RNA nanoparticles on the spheroid sprout formation. There was no effect on the CSL of the spheroids when treated with the core scaffold RNA nanoparticle 3WJ in the 3WJ series compared to the basal spheroids (*p* = 0.7425). With the conjugation of single or multiple anti-VEGF aptamers on the core scaffold 3WJ, the inhibitory effects on the CSL of the endothelial cells were enhanced, and the effects were related to the number of aptamers. The RNA nanoparticle with the single aptamer 3WJ-1V had the least effect among the three angiogenesis-inhibiting RNA nanoparticles, decreasing the CLS by 14.7% (*p* < 0.05 compared to spheroids treated with 3WJ). 3WJ-2V and 3WJ-3V further decreased the CLS of the endothelial spheroids to 673 and 471 µm, respectively (*p* < 0.0001 for both). The spheroids treated with 3WJ-3V had the lowest sprouting ability compared to those with 3WJ-1V and 3WJ-2V (*p* < 0.001 for both), which was similar to those inhibited by bevacizumab at the same concentration.

#### 3.2.2. SQR Series

**Figure 4:**
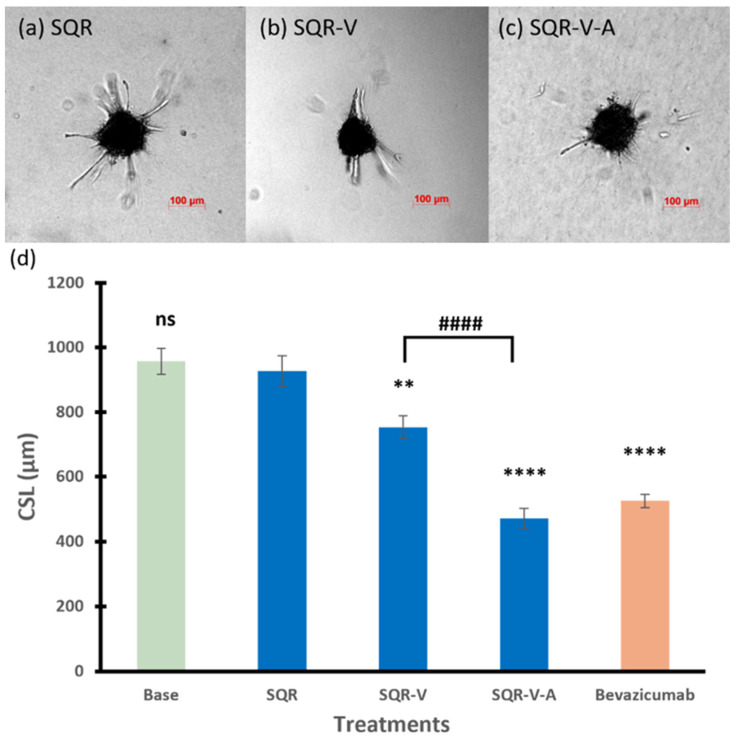
Representative images of spheroids treated with (**a**) SQR, (**b**) SQR-V, and (**c**) SQR-V-A. (**d**) The cumulative sprout length of spheroids treated with RNA nanoparticles in SQR series. Truncated mean ± SEM (n = 29–36). The symbol * indicates *t*-tests between the specific treatment and core scaffold RNA nanoparticle SQR, ns *p* > 0.05, ** *p* < 0.01, **** *p* < 0.0001; # indicates *t*-test between two specific treatments, #### *p* < 0.0001.

Similar to the 3WJ series, the core scaffold SQR in the SQR series had no effect on the spheroid sprouting activity (CSL of 927 µm). The RNA nanoparticle with single anti-VEGF aptamer SQR-V significantly inhibited the angiogenic sprouting of the spheroids, which decreased the CSL to 753 µm compared to those of spheroids treated with SQR (*p* < 0.01). Furthermore, the combination of anti-VEGF and anti-Ang2 reduced the CSL to 471 µm (*p* < 0.0001 compared to spheroids treated with SQR). Spheroids treated with SQR-V exhibited a decreased sprouting ability in terms of the sprout number, but there were still branch points observed (Figure 4b). The spheroid treated with SQR-V-A in Figure 4c had the lowest number and shortest length of sprouts and branching sprouts, showing that SQR-V-A had the highest inhibitory effects on tube formation of the spheroid in the SQR series.

#### 3.2.3. 8WJ Series

**Figure 5:**
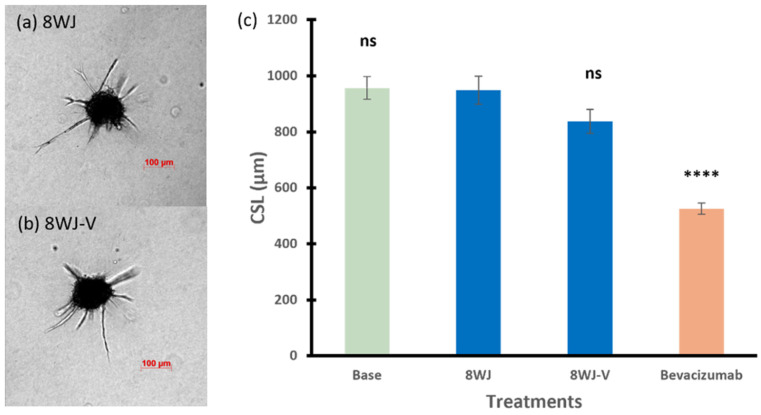
Representative images of spheroids treated with (**a**) 8WJ and (**b**) 8WJ-V. (**c**) The cumulative sprout length of spheroids treated with RNA nanoparticles in 8WJ series. Truncated mean ± SEM (n = 29–36). The symbol * indicates *t*-tests between the specific treatment and core scaffold RNA nanoparticle 8WJ, ns *p* > 0.05, **** *p* < 0.0001.

Figure 5a,b show the sprouting ability of the spheroids treated with 8WJ-V, with a single anti-VEGF aptamer (CSL of 838 µm), in which the CLS was slightly less than that of 8WJ (949 µm), but the difference is not statistically significant (*p* = 0.0999). In addition, there was no significant effect on the spheroid CLS compared to basal spheroids (*p* = 0.2059, ANOVA among base, 8WJ, and 8WJ-V).

#### 3.2.4. Among Series

**Figure 6:**
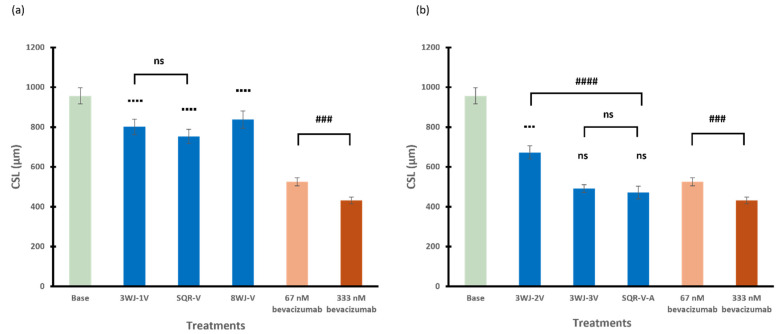
The cumulative sprout length of spheroids treated with RNA nanoparticles containing (**a**) single aptamer and (**b**) multiple numbers or types of aptamers at 67 nM. Truncated mean ± SEM (n = 29–36). The symbol ▪ indicates *t*-tests between the specific treatment and 67 nM bevacizumab, ns *p* > 0.05, ▪▪▪ *p* < 0.001, ▪▪▪▪ *p* < 0.0001; # indicates *t*-tests between two specific treatments, ns *p* > 0.05, ### *p* < 0.001, #### *p* < 0.0001.

The inhibitory effects of RNA nanoparticles in different series with either a single aptamer or multiple aptamers on spheroid sprouting (as CSL) were compared in Figure 6a and Figure 6b, respectively. In general, with only a single aptamer conjugated to the RNA nanoparticles, 3WJ-1V and SQR-V showed similar inhibitory effects (*p* = 0.3620) on the spheroids in terms of CSL. The spheroids treated with 3WJ-1V, SQR-V, and 8WJ-V showed significantly higher sprouting activities compared to those of bevacizumab (positive control) at the same concentration of 67 nM. As for the RNA nanoparticles with multiple aptamers, the inhibitory effect of 3WJ-2V, with two anti-VEGF aptamers, on spheroid sprouting was significant less than that of SQR-V-A with the combination of anti-VEGF and anti-Ang2 aptamers (*p* < 0.0001). The effect of 3WJ series nanoparticles on spheroid sprouting reached the same level as that of SQR-V-A when three anti-VEGF aptamers were conjugated to the core scaffold 3WJ (*p* = 0.5910), with an effect similar to that between 67 and 333 nM bevacizumab. In addition to the CSL analysis, Table 2 presents the numbers of sprouts per spheroid in selected treatments for comparison. The numbers of branching points per spheroid are also provided in the table. The effects of bevacizumab (positive control), VEGF (reference in model development), and RNA nanoparticles (3WJ-3V, SQR-V-A, and 8WJ-V at 67 nM) on tube formation and sprout branching are consistent with the increase in the length of sprouts in the CSL analysis. No decrease in the sizes of the spheroids was observed among the different treatments compared to the control.

### 3.3. Similar Results Between 3D and 2D Models

**Figure 7:**
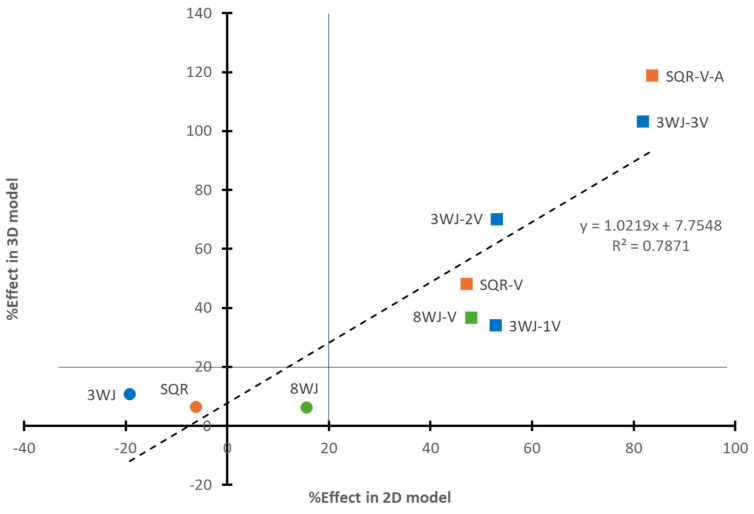
Relationship between %Effect of RNA nanoparticles based on bevacizumab at the same concentration in the 2D and 3D models. Symbols: circles represent core scaffold RNA nanoparticles; squares represent RNA nanoparticles with aptamers. Color: blue symbols represent RNA nanoparticles in 3WJ series; orange symbols represent RNA nanoparticles in SQR series; green symbols represent RNA nanoparticles in 8WJ series. The lines separate the regions of core scaffolds and RNA nanoparticles with aptamers.

The relationships between the %Effect observed in the 3D spheroid sprouting assay and the 2D WST-1 proliferation assay among different RNA nanoparticles in the 3WJ (3WJ, 3WJ-1V, 3WJ-2V, and 3WJ-3V), SQR (SQR, SQR-V, and SQR-V-A), and 8WJ series (8WJ and 8WJ-V) are shown in Figure 7. The linear least squares regression of the data showed a reasonable correlation between the %Effect of the 3D spheroid sprouting assay and the 2D WST-1 proliferation assay (R = 0.887), with a slope that is close to unity (1.022) and y-intercept of 7.8%. The left-bottom bracketed area (%Effect < 20%) in the figure illustrates the lack of inhibitory effects of the core scaffold nanoparticles in the present study. Two major differences were observed between the results in the 2D and 3D assay models. The %Effect of 3WJ-2V was different from that of 3WJ-1V in the 3D model but not in the 2D model. The core scaffold nanoparticles (3WJ, SQR, and 8WJ) had similar %Effect values in the 3D model, while they had %Effect values varying from −20% to 20% in the 2D model.

## 4. Discussion

Previously, the inhibitory effects of RNA nanoparticles with anti-angiogenesis aptamers, including anti-VEGF and anti-Ang2 aptamers, on cell proliferation were demonstrated via WST-1 cell proliferation assay on both HUVEC and human aortic endothelial cell (HAEC) models [12]. The WST-1 cell proliferation assay was a two-dimensional cell assay used to assess the RNA nanoparticles by cell number determination. To further investigate the antiangiogenic potential of these RNA nanoparticles, a 3D HUVEC spheroid sprouting assay was utilized in the present study. As the HAEC model was suggested to be less sensitive to angiogenic stimuli because of its shared origin with capillary structures [23], HUVEC was the only cell model selected for the 3D spheroid sprouting assay. For evaluating the RNA nanoparticles in the present study, a few modifications were made in the 3D spheroid sprouting assay based on the established 3D sprouting assay protocol [22,27,28]. A previous study showed that high abundance of growth factors in the serum-containing medium could lead to the lack of a bevacizumab-effect on HUVEC activity [29]. Thus, the medium for spheroid generation was modified to be a limited nutrient with VEGF as the only growth factor, and the final concentration of FBS added in the collagen matrix was decreased to 2%. To assess the feasibility of using the current 3D model to evaluate the RNA nanoparticles, bevacizumab, a VEGF-A targeting monoclonal antibody, was first evaluated. The results using the current model showed that 67 nM (10 µg/mL) bevacizumab significantly inhibited the spheroid sprouting ability, and its inhibitory effect was enhanced when the concentration increased to 333 nM (50 µg/mL). A previous study showed that VEGF could reach a plateau of its proangiogenic effects on HUVEC spheroids at 16 ng/mL (0.4 nM) [23]. Thus, VEGF at 0.008, 0.031, 0.125, and 0.5 nM were evaluated together with bevacizumab in the present study to demonstrate the sensitivity of the current 3D spheroid sprouting assay and the feasibility of the assay for evaluating the RNA nanoparticles. As expected, the proangiogenic effect of VEGF could be detected by the current 3D spheroid sprouting assay at a concentration as low as 0.031 nM, and it differed quantitively among various concentrations up to 0.5 nM. This suggests that the current model could be used to evaluate the inhibitory effect on endothelial cell tube formation and the antiangiogenic potential of the RNA nanoparticles.

In the present study, the inhibitory effects of RNA nanoparticles were evaluated at a concentration of 67 nM, which was the same as that of the positive control, bevacizumab. Bevacizumab is commonly used in the treatment of posterior eye neovascularization. Based on the clinical dose of bevacizumab treatment (1.25 mg) and the volume of human vitreous humor (around 4 mL), its initial concentration in the vitreous humor can be estimated (~2 µM). In a previous WST-1 cell proliferation study, similar effects were observed when HUVEC was treated with 100 nM and 2 µM bevacizumab [12]. There was also no significant difference between the effects of SQR-V-A at 10 and 100 nM in the same study. Therefore, 67 nM was the concentration examined in the present study.

For the studied core scaffold nanoparticles, they showed no effect on the spheroid sprouting. The effect of the RNA nanoparticles with anti-VEGF and anti-Ang2 aptamers were then investigated. When the number of conjugated anti-VEGF aptamers in the 3WJ series increased, 3WJ-3V exhibited the highest inhibition of the spheroid sprouting ability in the series. The difference between the inhibitory effects of 3WJ-1V, 3WJ-2V, and 3WJ-3V indicated that the increase in the number of aptamers resulted in higher inhibitory effects for the single-type anti-VEGF aptamer-conjugated RNA nanoparticles. Apart from multiple anti-VEGF aptamers, targeting VEGF and Ang2 could simultaneously provide higher antiangiogenic and antitumor effects compared to treatments targeting either VEGF or Ang2 alone in previous preclinical studies [30,31,32]. Consistent with these previous studies, SQR-V-A in the SQR series had significantly higher inhibitory effects than SQR-V on spheroid sprouting, suggesting that the combination of multiple types of therapeutic modules, i.e., anti-VEGF and anti-Ang2, could enhance the antiangiogenic ability of these RNA nanoparticles. In the 8WJ series, 8WJ-V, with only one therapeutic module conjugated to the RNA nanoparticle, had no significant effect on 3D spheroid sprouting. The other two single anti-VEGF aptamer-conjugated RNA nanoparticles, 3WJ-1V and SQR-V, decreased the sprouting ability of the spheroids, but their inhibitory effects were significantly less than bevacizumab at the same concentration. 3WJ-3V, with the highest number of anti-VEGF aptamers, and SQR-V-A, with the combination of anti-VEGF and anti-Ang2 aptamers, exhibited the highest inhibitory effects among the RNA nanoparticles tested in the present study, with similar effects to bevacizumab.

The effects of the RNA nanoparticles in the 3D spheroid sprouting assay were consistent with the results obtained using a 2D HUVEC cell proliferation assay in our previous study [12]. The similar results between the 3D and 2D models support the antiangiogenic effects of the RNA nanoparticles. The 3D and 2D models broadly agree with each other, despite some differences: (a) the lack of effects of the core scaffold nanoparticles was similar in the 3D model, while they varied from -20% to 20% in the 2D model; (b) the 3D model demonstrated the ability to differentiate the inhibitory effects of 3WJ-1V and 3WJ-2V, whereas in the 2D model, they showed similar effects; and (c) the 3D model showed equivalent inhibitory effects to bevacizumab at a lower nanoparticle concentration than the 2D model. In particular, it has been observed that the efficacy of the antiangiogenic RNA nanoparticles was highly dose-dependent, and 100 nM was the concentration of 3WJ-3V and SQR-V-A (RNA nanoparticles with the highest effects) necessary to reach the ~80% effect of bevacizumab in the 2D model [12]. However, these two RNA nanoparticles, at 67 nM, were observed to have equivalent effects to bevacizumab in the 3D spheroid sprouting assay. This suggests that the current 3D spheroid sprouting assay was sufficiently sensitive to evaluate and differentiate the effects of the RNA nanoparticles at a relatively low concentration. Moreover, the 3D spheroid sprouting assay could provide more perspectives from which to evaluate the inhibitory effects of RNA nanoparticles and their antiangiogenic potential. Although CLS was the key parameter selected for the quantitative analysis in the present study, the numbers and lengths of sprouts and branching sprouts were available for evaluation. For example, SQR-V-A and 3WJ-3V, the two most promising nanoparticles, could reduce the number of sprouts and the branching points at the same time.

In general, for the RNA nanoparticle with therapeutic anti-VEGF and anti-Ang2 aptamers, both the 3D and 2D models demonstrated similar inhibitory effects, supporting their utility in angiogenesis-related disease treatments. To assess the sensitivity of the 3D model and mimic the drug concentration after delivery, a relatively low concentration was selected in the present study. At this concentration, the nanoparticles were shown to have equivalent efficacy to bevacizumab, which suggests that the effective concentration of these nanoparticles in vivo could be lower than that projected by the 2D model. However, it should be noted that the 3D model is still an in vitro assay method. Further studies on animal models are required to confirm these effects.

It should also be noted that cell internalization of the RNA nanoparticles was previously observed using confocal fluorescence microscopy of the eye tissues (e.g., cornea, retinal pigment epithelium, and retina) obtained by sacrificing the animals at different time points after subconjunctival injection in vivo and through dissection of their eyes [12,33,34]. Cell internalization of the nanoparticles was not required to inhibit CSL and tube formation in the present study. The nanoparticles can bind to VEGF and Ang2 proteins extracellularly as the inhibition mechanism. Therefore, the evaluation of the cell internalization of the nanoparticles was not the present focus.

Furthermore, RNA nanoparticles are generally non-cytotoxic and non-immunogenic to tissues. The cytotoxicity of RNA nanoparticles has been studied previously, and they were demonstrated to be safe in animals [16,17,35,36]. In the present study, the sizes of the HUVEC spheroids did not decrease due to cell death after the nanoparticle treatments. This is consistent with the conclusions of the previous studies.

## 5. Conclusions

In the present study, the 3D HUVEC spheroid sprouting assay demonstrated the inhibitory effects of the RNA nanoparticles on endothelial cell tube formation and suggested the antiangiogenic potential of the nanoparticles at 67 nM. Among the RNA nanoparticles evaluated, 3WJ-3V and SQR-V-A had the highest inhibitory effects and were the most promising for anti-angiogenesis treatment. In addition, the present inhibitory results on 3D spheroid sprouting are consistent with those in the 2D WST-1 proliferation assay. This study suggests that the 3D spheroid sprouting assay is a reliable method to screen and evaluate the antiangiogenic or proangiogenic candidates or drugs.

## Figures and Tables

**Table 1:** RNA sequences of RNA nanoparticles. Lower-case letters indicate 2′-fluoro nucleotides; underlined letters indicate anti-VEGF or anti-Ang2 aptamer.

RNA Nanoparticles	Sequences
3WJ series:	
3WJ	A, 18 nt: 5′-uuG ccA uGu GuA uGu GGG-3′ B, 20 nt: 5′-ccc AcA uAc uuu Guu GAu cc-3′ C-AF647, 16 nt: 5′-GGA ucA Auc AuG GcA A-AF647-3′
3WJ-1V	A, 18 nt: 5′-uuG ccA uGu GuA uGu GGG-3′ B-VEGF, 47 nt: 5′-ccc AcA uAc uuu Guu GAu cc-cGG AAu cAG uGA AuG cuu AuA cAu ccG-3′ C-AF647, 16 nt: 5′-GGA ucA Auc AuG GcA A-AF647-3′
3WJ-2V	A-VEGF, 45 nt: 5′-uuG ccA uGu GuA uGu GGG-cGG AAu cAG uGA AuG cuu AuA cAu ccG-3′ B-VEGF, 47 nt: 5′-ccc AcA uAc uuu Guu GAu cc-cGG AAu cAG uGA AuG cuu AuA cAu ccG-3′ C-AF647, 16 nt: 5′-GGA ucA Auc AuG GcA A-AF647-3′
3WJ-3V	A-VEGF, 45 nt: 5′-uuG ccA uGu GuA uGu GGG-cGG AAu cAG uGA AuG cuu AuA cAu ccG-3′ B-VEGF, 47 nt: 5′-ccc AcA uAc uuu Guu GAu cc-cGG AAu cAG uGA AuG cuu AuA cAu ccG-3′ AF647-C-VEGF, 43 nt: 5′-AF647-GGA ucA Auc AuG GcA A-cGG AAu cAG uGA AuG cuu AuA cAu ccG-3′
SQR series:	
SQR	A, 46 nt: 5′-GAG ccG ucA Auc AuG GcA AGu Guc cGc cAu Acu uuG uuG cAc GcA c-3′ B, 46 nt: 5′-GAG cGu GcA Auc AuG GcA AGc GcA ucG cAu Acu uuG uuG cGA ccA A-3′ C-AF647, 46 nt: 5′-GAG Guc GcA Auc AuG GcA AcG AuA GAG cAu Acu uuG uuG Gcu GGA G-AF647-3′ D, 46 nt: 5′-GAc cAG ccA Auc AuG GcA AuA uAc AcG cAu Acu uuG uuG AcG GcG G-3′ E, 88 nt: 5′-GGA cAc uuG ucA uGu GuA uGc GuG uAu Auu Guc AuG uGu AuG cuc uAu cGu uGu cAu GuG uAu GcG AuG cGc uuG ucA uGu GuA uGG c-3′
SQR-V	A, 46 nt: 5′-GAG ccG ucA Auc AuG GcA AGu Guc cGc cAu Acu uuG uuG cAc GcA c-3′ B-VEGF, 73 nt: 5′-GAG cGu GcA Auc AuG GcA AGc GcA ucG cAu Acu uuG uuG cGA ccA A-cGG AAu cAG uGA AuG cuu AuA cAu ccG-3′ C-AF647, 46 nt: 5′-GAG Guc GcA Auc AuG GcA AcG AuA GAG cAu Acu uuG uuG Gcu GGA G-AF647-3′ D, 46 nt: 5′-GAc cAG ccA Auc AuG GcA AuA uAc AcG cAu Acu uuG uuG AcG GcG G-3′ E, 88 nt: 5′-GGA cAc uuG ucA uGu GuA uGc GuG uAu Auu Guc AuG uGu AuG cuc uAu cGu uGu cAu GuG uAu GcG AuG cGc uuG ucA uGu GuA uGG c-3′
SQR-V-A	A, 46 nt: 5′-GAG ccG ucA Auc AuG GcA AGu Guc cGc cAu Acu uuG uuG cAc GcA c-3′ B-VEGF, 73 nt: 5′-GAG cGu GcA Auc AuG GcA AGc GcA ucG cAu Acu uuG uuG cGA ccA A-cGG AAu cAG uGA AuG cuu AuA cAu ccG-3′ C-AF647, 46 nt: 5′-GAG Guc GcA Auc AuG GcA AcG AuA GAG cAu Acu uuG uuG Gcu GGA G–AF647-3′ D-Ang2, 87 nt: 5′-GAc cAG ccA Auc AuG GcA AuA uAc AcG cAu Acu uuG uuG AcG GcG G-GAGGAc GAu GcG GAc uAG ccu cAu cAG cuc AuG uGc ccc uc-3′ E, 88 nt: 5′-GGA cAc uuG ucA uGu GuA uGc GuG uAu Auu Guc AuG uGu AuG cuc uAu cGu uGu cAu GuG uAu GcG AuG cGc uuG ucA uGu GuA uGG c-3′
8WJ series:	
8WJ	AF647-A, 42 nt: 5′-AF647-GAG uAu AuG uuA GGc cuG GGu GAG ucc uuG cGu cuu cuA ccG-3′ B, 42 nt: 5′-cGG uAG AAG AcG cAA GGA cuu Gcu AGu uGu GGu Acu Guu ccc-3′ C, 42 nt: 5′-GGG AAc AGu Acc AcA Acu AGu Guc ccG GGA uAG GGA cAu AcA-3′ D, 42 nt: 5′-uGu AuG ucc cuA ucc cGG GAu Gcu ccG cAu GAu GAA uAc AGc-3′ E, 42 nt: 5′-Gcu GuA uuc Auc AuG cGG AGu GGG cAu uGG GAu cGu AuG AGc-3′ F, 42 nt: 5′-Gcu cAu AcG Auc ccA AuG ccu GAA cAA AcA GAG cAA Gcc ucc-3′ G, 42 nt: 5′-GGA GGc uuG cuc uGu uuG uuu GcG cGA uuu ccG cGu uAc AcA-3′ H, 42 nt: 5′-uGu GuA AcG cGG AAA ucG cGu Gcc cAG Gcc uAA cAu AuA cuc-3′
8WJ-V	AF647-A, 42 nt: 5′-AF647-GAG uAu AuG uuA GGc cuG GGu GAG ucc uuG cGu cuu cuA ccG-3′ VEGF-B, 69 nt: 5′-cGG AAu cAG uGA AuG cuu AuA cAu ccG-cGG uAG AAG AcG cAA GGA cuu Gcu AGu uGu GGu Acu Guu ccc-3′ C, 42 nt: 5′-GGG AAc AGu Acc AcA Acu AGu Guc ccG GGA uAG GGA cAu AcA-3′ D, 42 nt: 5′-uGu AuG ucc cuA ucc cGG GAu Gcu ccG cAu GAu GAA uAc AGc-3′ E, 42 nt: 5′-Gcu GuA uuc Auc AuG cGG AGu GGG cAu uGG GAu cGu AuG AGc-3′ F, 42 nt: 5′-Gcu cAu AcG Auc ccA AuG ccu GAA cAA AcA GAG cAA Gcc ucc-3′ G, 42 nt: 5′-GGA GGc uuG cuc uGu uuG uuu GcG cGA uuu ccG cGu uAc AcA-3′ H, 42 nt: 5′-uGu GuA AcG cGG AAA ucG cGu Gcc cAG Gcc uAA cAu AuA cuc-3′

**Table 2:** The numbers of sprouts per spheroid and branching points per spheroid and the size of the spheroids in representative treatments. Truncated mean ± SEM (n = 29–36).

Treatment	Sprout Number	Branching Point Number	Spheroid Size (Diameter, µm)
Control/base	11.8 ± 0.5	2.3 ± 0.3	112.1 ± 1.0
0.5 nM VEGF	15.9 ± 0.7	5.7 ± 0.6	114.0 ± 0.9
67 nM bevacizumab	8.2 ± 0.3	0.47 ± 0.12	112.0 ± 1.0
333 nM bevacizumab	7.7 ± 0.3	0.50 ± 0.12	111.4 ± 1.0
3WJ-3V	8.5 ± 0.4	0.68 ± 0.13	113.0 ± 1.3
SQR-V-A	8.1 ± 0.5	0.51 ± 0.13	114.0 ± 1.0
8WJ-V	11.6 ± 0.4	1.00 ± 0.18	113.2 ± 1.1

## Data Availability

The data presented in this paper can be made available upon request to the corresponding author.
